# Targeted Treatments of Bone Metastases in Patients with Lung Cancer

**DOI:** 10.3389/fonc.2014.00146

**Published:** 2014-06-16

**Authors:** Vera Hirsh

**Affiliations:** ^1^McGill University Health Centre, Royal Victoria Hospital, Montreal, QC, Canada

**Keywords:** bone metastases, denosumab, zoledronic acid, NSCLC, biomarkers, skeletal-related events, effect on pain and survival

## Abstract

Until now ~30–40% of patients with advanced lung cancer develop bone metastases, but as the newer therapies are extending survival, the chance of developing bone metastases increases. Bone metastases cause skeletal-related events (SREs) such as pathologic fractures, spinal cord compression, radiation therapy or surgery to bone, or hypercalcemia, which can have debilitating consequences affecting patients’ health-related quality of life (HR-QOL) and performance status (PS). Poor PS then prevents the patients to receive further lines of treatments, which are available today. SREs are associated with increased economic costs. In one clinical trial, the median time to first SRE was only 5 months. Early detection of bone metastases can prevent SREs and avoid inappropriate implementation of major surgery or chemoradiation therapy. With the new generation bisphosphonate zoledronic acid (ZA) or denosumab (anti-RANKL activity), one can reduce the number of patients who experience SREs, decrease the annual incidence of SREs and delay the median time to first SRE. These agents are effective even after the onset of SREs. They are well tolerated, with manageable side effects. The biochemical markers of bone metabolism especially N-telopeptide of type I collagen and bone specific alkaline phosphatase (BALP) can be both prognostic and predictive markers for the patients with bone metastases from non-small cell lung cancer (NSCLC). Anticancer activity of ZA and denosumab further supports their use as soon as bone metastases are diagnosed in patients with NSCLC. Further trials will inform us about the efficacy of these agents for prevention of bone metastases and even about possible effects on visceral metastases.

## Introduction

Approximately 30–40% of patients with lung cancer develop bone metastases (1), which can lead to skeletal-related event (SREs) such as pathologic fractures, spinal cord compression, radiation therapy or surgery to bone, or hypercalcemia. These SREs can affect the patient’s health-related quality of life (HR-QOL). Bone metastases are the most common cause of cancer-associated pain in patients with advanced malignancies (2). The bone pain associated with bone metastases often requires palliative radiation therapy. Pathologic fracture, which may require surgery, spinal cord compression, and hypercalcemia of malignancy (HCM) can be life-threatening. In a large prospective trial, pathologic fractures were significantly and negatively correlated with survival among 460 patients with bone metastases from solid tumors, including breast, prostate, kidney, and lung cancers (3). SREs not only cause increased morbidity and deterioration of performance status (PS), but also increased economic costs (4), thus SRE prevention will not only decrease patient morbidity, improve HR-QOL, but will also be associated with decreased use of health care resources. The need to focus on bone metastases and their sequelae is heightened as the survival of patients with non-small cell lung cancer (NSCLC) increases with the newer therapies. In one clinical trial, median time to first SRE in patients with NSCLC was 5 months only (5). To prevent SREs, preserve patients’ QOL, good PS, and functional independence are of great importance and will allow patients to receive all the lines of therapies now available.

## Pathophysiology of Bone Metastases

The release of growth factors from the bone matrix during osteoclast-mediated osteolysis is conducive to the development of metastatic lesions (6). In osteolytic lesions, factors secreted by tumor cells induce osteoclast recruitment and activation, leading to increased osteolysis (7). Elevated osteolysis decreases bone integrity, can cause bone pain and the release of minerals from the bone matrix, resulting in HCM (8). Bone resorption releases growth factors that stimulate tumor growth and increase of osteoclast-stimulating factors (9). In contrast, tumor cells in osteoblastic lesions secrete factors that stimulate osteoblasts, which are responsible for the formation of new bone tissue (osteogenesis). Levels of osteolysis are enhanced in response to increased osteogenesis, releasing growth factors from the bone matrix (7). Osteoblastic lesions may also contain a strong osteolytic component that can decrease bone integrity (9, 10). Aberrant new bone formation in osteoblastic lesions produces new bone tissue that is abnormal, malformed, and does not add to the overall bone strength (9, 11).

## Early Detection of Bone Metastases

The incorrect staging of patients with NSCLC can result in suboptimal treatment decisions such as major surgery or an aggressive chemoradiation without hope for a curative outcome.

Recently, PET scanning for accurate staging of NSCLC has been recognized as a valuable tool by the National Comprehensive Cancer Network (12). Fluorine-18 deoxyglucose (FDG)-PET scans for the detection of bone metastases in NSCLC have been shown to have a higher specificity compared with bone scans (~90 versus 70%, respectively) (13, 14) and a much lower rate of false negatives (6 versus 39%, respectively) (15). The sensitivity of FDG-PET and bone scans for the detection of bone metastases from NSCLC was comparable after appropriate follow-up imaging (13, 14).

## Clinical Implications of Bone Metastases – Bisphosphonates, Zoledronic Acid

Bisphosphonates are pyrophosphate analogs that are deposited at sites of bone remodeling. They bind to bone mineral surfaces and are ingested by osteoclasts wherein they inhibit osteolysis (16). Early bisphosphonates i.e., etidronate, clodronate, demonstrated efficacy for the treatment of HCM, but these agents are weak with limited utility in the oncology setting (16).

The introduction of a nitrogen group to the bisphosphonate backbone resulted (17) in increased potency and a different cellular target: farnesyl diphosphonate synthase, a key enzyme in the mevalonate pathway. These bisphosphonates inhibit protein prenylation and RAS signaling in osteoclasts, thereby inducing apoptosis (18). Zoledronic acid consistently achieved the greatest antiresorptive efficacy among the bisphosphonates tested in preclinical assays in human cancer cell lines and animal models (19, 20).

Regulatory approval for zoledronic acid (ZA) in patients with any solid tumors was based on results from a phase III randomized, placebo-controlled trial in which 773 patients with bone metastases from solid tumors other than breast or prostate cancer received ZA (4 or 8 mg) or placebo via 15 min intravenous infusion every 3 weeks for up to 21 months (5). Among the 507 patients randomized to the 4 mg ZA or placebo group of this trial, 249 had NSCLC and 36 had small cell lung cancer (SCLC).

**Figure 1:**
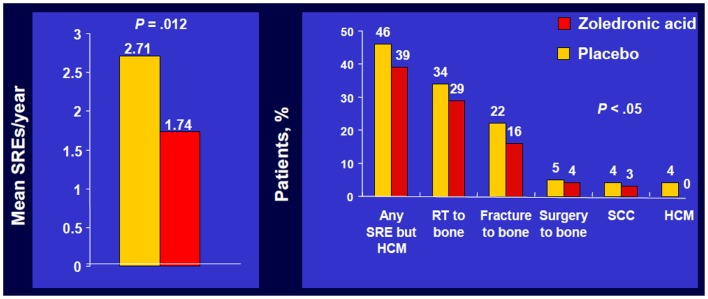
**Zoledronic Acid reduced percentage of patients with each SRE**. Phase III trial of patients with bone metastases from NSCLC/OST who received ZOL or placebo every 3 weeks for up to 21 months. Approximately 50% of patients had NSCLC; ~7% of patients had SCLC. SRE, skeletal-related event; mets, metastases; NSCLC, non-small cell lung cancer; OST, other solid tumors; RT, radiotherapy; SCC, spinal cord compression; HCM, hypercalcemia of malignancy. Data from Rosen et al. (5).

In the overall trial population, ZA significantly reduced the number of patients who experienced at least one SRE, including HCM, 39 versus 48% with placebo, *p* = 0.039, and reduced the proportion of patients who experienced each type of SRE (Figure 1) (5).

Zoledronic acid also significantly decreased the annual incidence of SREs, 1.74 versus 1.71 per year for placebo, *p* = 0.012 and significantly delayed the median time to first SRE compared with placebo (236 versus 155 days, respectively, *p* = 0.009) (5). A multiple event analysis using a robust Andersen–Gill model was performed for the overall population. This analysis takes into account not only the number of SREs but also the timing between the SREs, thereby providing a sensitive comparison of the ongoing risk of SREs between two treatment groups.

Zoledronic acid reduced the risk of SREs by 31% versus placebo in the overall trial population (relative risk, RR = 0.693, *p* = 0.003). Many patients with lung cancer are diagnosed only after the first SRE. However, pre-existing skeletal morbidity does not preclude the benefits of subsequent therapy with ZA. Indeed, patients who have already experienced an SRE are at especially high risk for subsequent events. In an exploratory analysis of the ZA phase III trial in patients with NSCLC and other solid tumors, patients with a history of SRE before study entry had a 41% increased risk of experiencing an on-study SRE compared with patients with no history of prior SRE (*p* = 0.036) (21). In patients with a prior SRE, ZA produced a significant 31% reduction in the risk of developing an on-study SRE compared with placebo in a robust Andersen–Gill multiple event analysis, *p* = 0.009, and significantly reduced the skeletal morbidity rate, 1.96 versus 2.81 events per year for placebo, *p* = 0.030 (21).

Furthermore, ZA significantly prolonged the median time to first SRE on study by ~4 months compared with placebo in this prior-SRE cohort (215 versus 106 days, respectively, *p* = 0.011). Benefits were also seen in the subset of patients who had not experienced a prior SRE, but without a statistical significance because of lack of the statistical power. This study suggests that ZA is effective and provides benefits even after the onset of SREs.

The most commonly reported adverse events (AEs) for ZA and placebo during the trial were bone pain including infusion of ZA-related pain (48 and 58%, respectively), nausea (47 and 32%, respectively), and dyspnea (45 and 30%, respectively) (22). There was no significantly lower incidence of palliative radiotherapy to bone in the 4 mg ZA group versus placebo (23). There were no grade 4 increases in serum creatinine in the NSCLC stratum. Monitoring of renal function and oral health during bisphosphonate therapy is recommended to avoid uncommon, but potentially serious AEs (24, 25). Because all intravenous bisphosphonates are cleared by the kidneys, renal function, and hydration status should be determined before each infusion to ensure renal safety. Reduced starting dose of ZA is recommended for patients with impaired renal function (26).

Osteonecrosis of the jaw (ONJ) has been reported as an uncommon event in patients receiving bisphosphonates and is characterized by exposed bone in the maxillofacial area with no evidence of healing after 6 weeks of appropriate dental care in the absence of metastatic disease or radiation to the jaw (25). The reports using the data obtained from retrospective analyses and reviews of medical records databases suggest that the frequency of ONJ in patients with malignant bone disease may be between 0.7 and 12.6% (27–29).

This wide range in ONJ frequency is likely due to variability in preventive dental measures before and during bisphosphonate therapy, variations in the duration of bisphosphonate treatment, and geographic differences. Preventive dental measures and appropriate oral hygiene have been identified that can significantly reduce the incidence of ONJ during bisphosphonate therapy (25, 30–32). A pilot study in patients with active ONJ lesions found that local application of a medical ozone oil suspension led to complete ONJ resolution (33).

## Zoledronic Acid and Biochemical Markers

**Figure 2:**
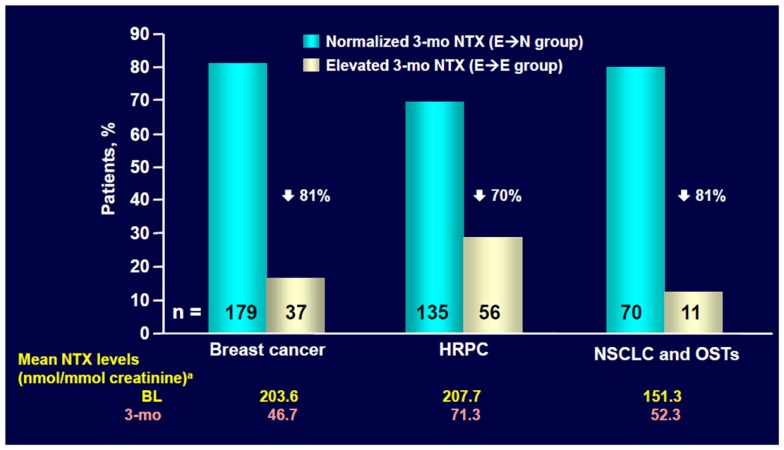
**ZOL normalized NTX levels within 3 months in most patients with elevated baseline NTX**. NTX, N-telopeptide of type I collagen; HRPC, hormone-refractory prostate cancer; NSCLC, non-small cell lung cancer; OST, other solid tumors; BL, baseline. Data from Lipton et al. (35).

In a subset of patients with NSCLC or other solid tumors in the placebo group (238 patients), urinary levels of the bone resorption marker N-telopeptide of type I collagen (NTX) and the serum bone formation marker bone specific alkaline phosphatase (BALP) were assessed approximately every 3 months (34). High NTX levels (≥100 nmol/mmol creatinine) at baseline were associated with an increased risk of first SRE (RR = 1.85, *p* = 0.076) and bone disease progression (RR = 1.76, *p* = 0.029) compared with patients with low NTX levels (<100 nmol/mmol creatinine, Figure 2) (34). Moreover, compared with patients with low NTX levels, patients with high NTX levels had a more than threefold increased risk of death (RR = 3.03, *p* < 0.001) and a 5-month reduction in median survival (3.2 versus 8.2 months for patients with low baseline NTX levels) (34). Patients with high baseline BALP levels (≥146 IU/L) also had statistically significant increases in risk of disease progression (RR = 1.77, *p* = 0.005) and death (RR = 1.53, *p* = 0.003) compared with patients with low BALP levels (<146 IU/L) (34).

Exploratory analysis of the ZA phase III clinical trial database (36) showed that ZA reduced mean urinary NTX levels within 3 months in patients with bone metastases from NSCLC and other solid tumors who had bone marker assessment (*n* = 204) (35). ZA also significantly reduced the RR of death by 35% versus placebo (RR = 0.650, *p* = 0.024) among patients with NSCLC and high baseline NTX levels (NTX ≥ 64 nmol/mmol creatinine, *n* = 144) (37).

Differences in survival between the ZA and placebo groups did not reach statistical significance in the normal baseline NTX subset, consistent with the lower risks of SREs and death that have been reported for that subset (34, 37).

This benefit could result from reduced osteolysis, resulting in less release of growth factors from the bone matrix, reduced SRE rate or possibly also from direct and indirect antitumor effects of ZA i.e., increased apoptosis, synergism with chemotherapy, antiangiogenesis, and stimulation of immune system.

## Anticancer Activity of Zoledronic Acid

There is a preclinical evidence that ZA can inhibit proliferation and induce apoptosis in a broad range of human cancer cell lines (16, 38) ZA also exerts antitumor synergy with chemotherapy agents in the A549 lung cancer cell line (39, 40). In murine lung cancer cell line, ZA inhibited the growth of these tumors and mice treated with ZA survived significantly longer than the untreated mice (*p* < 0.05) (41).

Multiple effects may contribute to the antitumor activity of ZA that has been reported in preclinical models (42). In addition to direct antitumor effects, nitrogen-containing bisphosphonates appear to have immunomodulatory properties especially with regard to γδ T cells, a subset of T cells that plays a role in immunosurveillance for malignancies. In an *in vitro* model, ZA induced maturation and upregulated co-stimulating surface receptor expression (e.g., CD 40, CD 80, CD 83) on peripheral γδ T cells (43). In addition, bisphosphonates have been shown to activate the cytolytic activity of γδ T cells and therefore, may enhance the antitumor immune response (44).

There are ongoing clinical studies in patients with NSCLC evaluating the efficacy of ZA both for prevention of bone metastases and for antitumor activity.

## Denosumab and Anti-RANKL Activity

Denosumab is a fully human monoclonal antibody that binds to and neutralizes RANKL (receptor activator of nuclear factor kappa-B ligand) thereby inhibiting osteoclast function and preventing generalized bone resorption and local bone destruction.

It is hypothesized that tumor cells in the bone lead to increased expression of RANKL on osteoclasts and their precursors. RANKL is an essential mediator of osteoclast function, formation, and survival (45–47). Excessive RANKL-induced osteoclast activity results in resorption and local bone destruction with evidence of elevated levels of bone turnover markers, leading to SREs (34, 36).

Denosumab has been studied in two phase II trials of patients with bone metastases in advanced cancer and in one phase II trial with myeloma (48–50). These studies demonstrated that treatment with denosumab at doses ranging from 30 to 180 mg administered every 4 or 12 weeks was associated with a rapid and sustained suppression of bone turnover markers and delay of SREs similar to that seen with i.v. bisphosphonates.

In a randomized, double-blind phase III trial of denosumab versus ZA, in the treatment of bone metastases in patients with advanced cancer (excluding breast and prostate cancer) or multiple myeloma, 1779 patients were enrolled onto study, 890 patients analyzed on ZA, 886 on denosumab (51). Baseline characteristics were well balanced (Table 1). The primary endpoint was time to first on-study SRE comparing denosumab with ZA for non-inferiority. Secondary efficacy endpoints were to be evaluated only if non-inferiority was demonstrated, and were superiority tests comparing denosumab and ZA for time to first on-study SRE and time to first and subsequent SRE by multiple event analysis. A subsequent SRE was defined as an event occurring ≥21 days after the previous SRE.

**Table 1:** **Baseline characteristics**.

Characteristic, *n* (%) or median	Zoledronic acid (*n* = 890)	Denosumab (*n* = 886)
Male	552 (62)	588 (66)
Age (years)	61	60
Primary tumor type
Non-small cell lung cancer	345 (39)	343 (39)
Multiple myeloma	93 (10)	86 (10)
Other	452 (51)	457 (52)
ECOG performance status of 0 or 1	728 (82)	748 (84)
Time from first bone metastasis to randomization (months)	2	2
Previous SRE	446 (50)	440 (50)
Presence of visceral metastases	448 (50)	474 (53)

*See Ref. (52)*.

The median number of doses was seven for ZA and seven for denosumab with cumulative drug exposure of 651.9 patient-years for ZA and 675.3 patient-years for denosumab. Median time on study was ~7 months.

**Figure 3:**
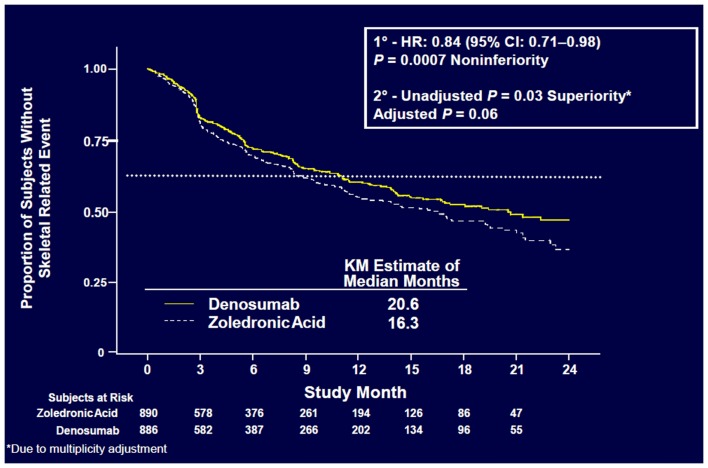
**Time to first on-study SRE (52)**.

**Figure 4:**
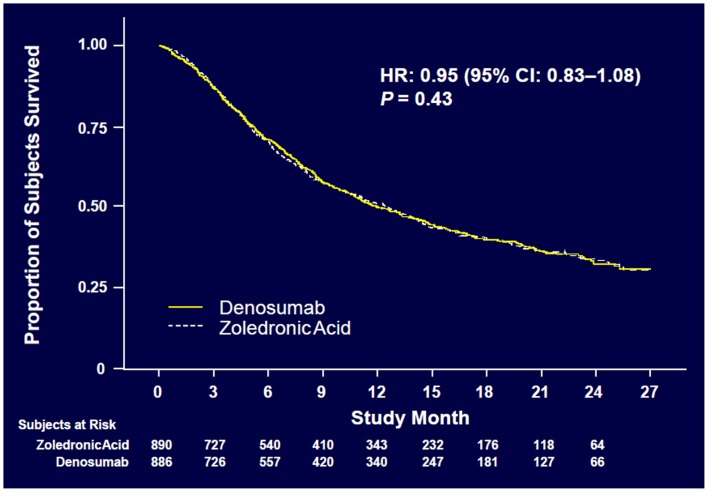
**Overall survival (52)**.

**Figure 5:**
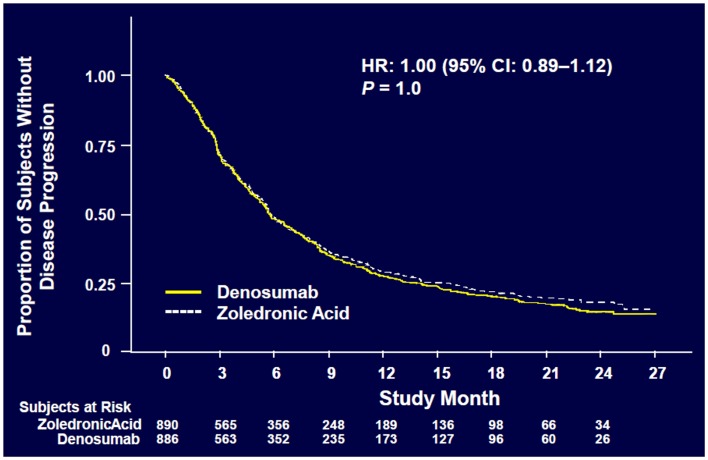
**Overall disease progression (52)**.

Denosumab was non-inferior to ZA in delaying time to first on-study SRE (HR = 0.84, *p* = 0.0007) representing 16% reduction in hazard (Figure 3). The median time to first on-study SRE was 20.6 months for denosumab and 16.3 months for ZA. The test for superiority for time to first SRE showed *p* = 0.06 and therefore did not reach statistical significance. Time to first and subsequent SREs (multiple events) analysis demonstrated a rate ratio of 0.90 for denosumab compared with ZA, *p* = 0.14, which was not statistically significant. Overall survival (HR = 0.95, *p* = 0.43) and disease progression (HR = 1.00, *p* = 1.0) were similar between treatment groups (Figures 4 and 5).

The effect of denosumab on time to first on-study SRE relative to ZA by tumor stratification factors resulted in an HR = 0.84 for NSCLC, *p* = 0.20; 1.03 for myeloma, *p* = 0.89, and 0.79 for other solid tumors, *p* = 0.04. An *ad hoc* analysis examining overall survival demonstrated an HR = 0.79 for NSCLC, 2.26 for myeloma, and 1.08 for other solid tumors.

Patients in both arms experienced similar rates of AEs (Table 2). Rates of serious AEs are 13.4% for ZA versus 14.6% for denosumab. New primary malignancy occurred in three patients (0.3%) receiving ZA and in five patients (0.6%) receiving denosumab.

**Table 2:** **Adverse events of interest**.

Event, *n* (%)	Zoledronic acid (*n* = 878)	Denosumab (*n* = 878)
Infectious AEs	349 (39.7)	358 (40.8)
Infectious serious AEs	118 (13.4)	128 (14.6)
Acute phase reaction (first 3 days)	127 (14.5)	61 (6.9)
Potential renal toxicity AEs^a^	96 (10.9)	73 (8.3)
Renal failure	25 (2.8)	20 (2.3)
Acute renal failure	16 (1.8)	11 (1.3)
Cumulative rates of ONJ*	11 (1.3)	10 (1.1)
Year 1	5 (0.6)	4 (0.5)
Year 2	8 (0.9)	10 (1.1)
New primary malignancy	3 (0.3)	5 (0.6)

*^a^ Includes blood creatinine increased, renal failure, renal failure acute, proteinuria, blood urea increased, renal impairment, urine output decreased, anuria, oliguria, azotemia, hypercreatininemia, creatinine renal clearance decreased, renal failure chronic, blood creatinine abnormal*.

*p = 1.0.

*No neutralizing anti-denosumab antibodies were detected*.

*See Ref. (52)*.

Adverse events of hypocalcemia occurred more frequently with denosumab (10.8% denosumab, 5.8% ZA). In general, the clinical consequences of hypocalcemia were not observed. Centrally determined grade 3 and 4 decreases in albumin-adjusted calcium values were reported in 9 patients (1%) receiving ZA and 20 patients (2.3%) receiving denosumab. IV calcium was administered on study to 2.7% of patients receiving ZA and 5.7% receiving denosumab.

Positive adjudicated ONJ occurred with cumulative incidence rates in the ZA and denosumab groups of 0.6 and 0.5% at 1 year, respectively, 0.9 and 1.1% at 2 years, and 1.3 and 1.1% at 3 years (*p* = 1.0).

Adverse events associated with acute phase reactions within the first 3 days after dose 1 occurred in 14.5% of patients receiving ZA versus 6.9% receiving denosumab. Most frequent reactions were pyrexia, arthralgia, and fatigue. One hundred fifty-two patients (17.3%) on ZA required dose adjustments to levels lower than 4 mg and doses were withheld because of elevated serum creatinine in 78 patients (8.9%). No dose adjustments or dose withholding for renal function were required for denosumab. Despite appropriate adjustments of the ZA dosing regimen for renal function, there was an evidence of an excess of renal AEs with ZA. Denosumab has no limitations with respect to renal impairment as it is a monoclonal antibody and is eliminated by intracellular catabolism in phagocytes, with no evidence of renal effects (53, 54).

## Bone Turnover Biomarkers – Denosumab Versus Zoledronic Acid

Patients treated with denosumab experienced a greater suppression of bone turnover markers than with ZA. Between baseline and study week 13 levels of urinary NTX/Cr decreased by a median of 76% for denosumab (*n* = 546) and 65% for ZA (*n* = 543), *p* < 0.001 and BALP decreased by 37% for denosumab (*n* = 578) and 29% for ZA (*n* = 581), *p* < 0.001.

## Exploratory Analysis of Overall Survival in Lung Cancer

**Figure 6:**
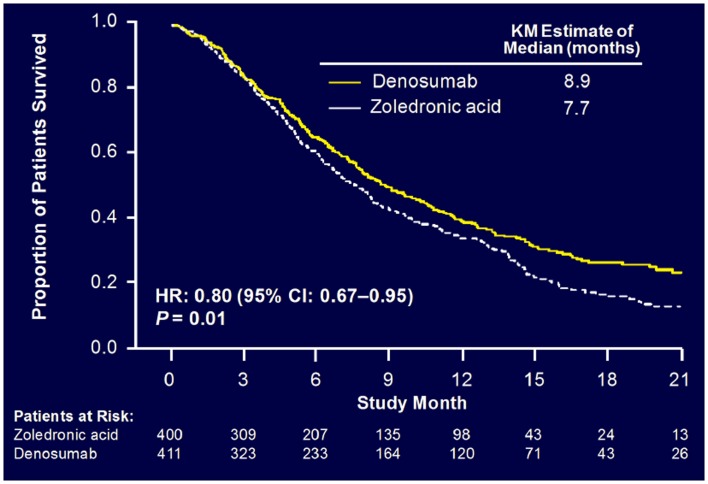
**Overall survival: patients with lung cancer**.

**Figure 7:**
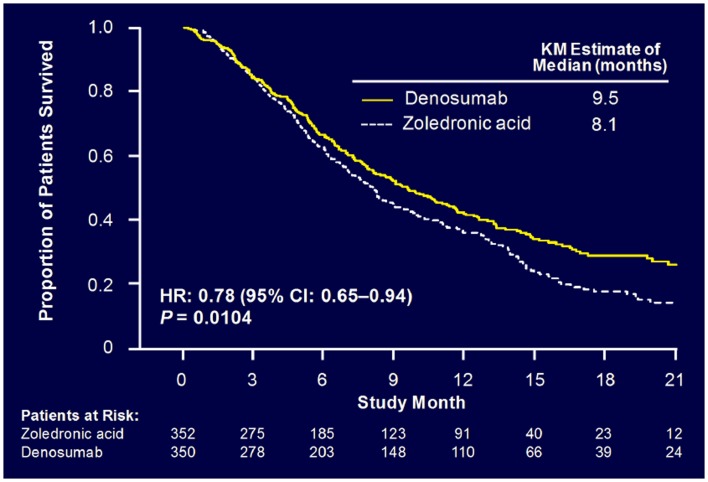
**Overall survival: patients with NSCLC**.

Sub-analysis of 811 patients with any lung cancer showed that denosumab was associated with significantly improved overall median survival compared with ZA, with a difference of 1.2 months (KM median = 8.9 versus 7.7 months, HR = 0.80, *p* = 0.01) (Figure 6) (55). Denosumab continued to show a significant survival advantage over ZA when overall survival was adjusted for relevant baseline covariates (age, sex, time from diagnosis of primary cancer to first evidence of metastasis or the first bone metastasis, visceral metastasis, and ECOG status) and stratified by the randomization stratification factors (previous SRE and systemic anticancer therapy), HR = 0.81, *p* = 0.01. In patients with visceral metastases (231 in denosumab group and 233 in ZA group), denosumab was also associated with improved overall median survival with a difference of 1.2 months (KM median = 7.7 versus 6.4 months, HR = 0.79, *p* = 0.03). Denosumab was associated with significantly improved survival in patients with NSCLC with a difference of 1.5 months (KM median = 9.5 versus 8.1 months, HR = 0.78, *p* = 0.01) (Figure 7).

Explanation for the longer survival with the denosumab treatment in these lung cancer patients includes both direct and indirect effects on tumor cells. An indirect effect may derive from the symbiotic relationship between tumor cells and the bone marrow microenvironment in which both bone destruction and tumor growth are promoted. Tumor cells secrete various factors that stimulate production of RANKL (45). The increased expression of RANKL in the tumor environment leads to increased formation, activation, and survival of osteoclasts and results in osteolytic lesions (56). Osteolysis then results in the release of growth factors derived from bone (45, 57).

These growth factors increase the production of parathyroid hormone-related protein or promote tumor growth directly (45). Bone destruction increases local extracellular calcium concentrations, which have also been shown to promote tumor growth and the production of parathyroid hormone-related protein (57). Denosumab may indirectly affect skeletal tumor progression by targeting osteoclasts and disrupting the interaction between tumor cells and the bone microenvironment. RANKL inhibition has been shown to reduce bone lesions/osteolysis, and skeletal tumor burden in a model of NSCLC (58) and to enhance antitumor efficacy of other therapies on skeletal tumors (59, 60).

Another hypothesis is that denosumab may improve survival by directly inhibiting RANKL on RANK-expressing tumor cells, which has been demonstrated for breast cancer cells in vivo (61) and for a number of tumor cell lines (including lung cancer cells) in vitro (62). RANKL inhibition may have a direct antineoplastic effect on lung cancer cells via apoptosis or anti-migration activity (63). The hypothesis of mechanism of anticancer activities, which inhibit RANKL or RANK-expressing tumor cells has been described in more detail in the review article of Peters and Meylan (64). These findings warrant further prospective clinical investigations, denosumab might have anticancer effects beyond the skeleton (65).

## Promising New Bone Targeting Agents

Targeting bone agents in the early stage of investigation in NSCLC are Dasatinib (i.e., anti-src activity) (66), ACE-011 (Sotatercept – Activin TRAP) (67, 68), Cabozantinib (anti-RET agent) (69), and Radium 223 (targeted alpha emitter) (70).

## Conclusion

In the palliative group of patients with metastatic lung cancer, the HR-QOL is extremely important. Preserving a good PS, which enables these patients to receive all the available lines of treatment for metastatic NSCLC is also desirable.

Early identification of bone metastases and management of SREs have become crucial for maintaining QOL and containing healthcare costs throughout the patient’s care. The identification of risk factors for skeletal metastases in patients with NSCLC will help us to implement early treatment to prevent or delay the onset of debilitating SREs.

The safety profile for ZA and denosumab is similar but subcutaneous administration of denosumab offers advantages over intravenous administration with no need for renal monitoring. Denosumab is associated with fewer acute phase reactions, but has a higher incidence of hypocalcemia. ONJ is similar for both agents. Thus both agents are a reasonable option for targeted bone therapy.

Future trials are needed to inform us about efficacy of these agents for prevention of bone metastases and effects on visceral metastases, too, thus contributing to a longer survival in patients with metastatic NSCLC.

## Conflict of Interest Statement

The author declares that the research was conducted in the absence of any commercial or financial relationships that could be construed as a potential conflict of interest.
